# Transcatheter aortic valve replacement in a bicuspid aortic valve with membranous interventricular septum aneurysm communicating with aortic root: a case report

**DOI:** 10.1093/ehjcr/ytae523

**Published:** 2024-10-04

**Authors:** Ziwei Xi, Jing Yao, Guangyuan Song

**Affiliations:** Interventional Center of Valvular Heart Disease, Beijing Anzhen Hospital, Capital Medical University, No. 2 Anzhen Road, Chaoyang District, Beijing 100029, China; Interventional Center of Valvular Heart Disease, Beijing Anzhen Hospital, Capital Medical University, No. 2 Anzhen Road, Chaoyang District, Beijing 100029, China; Interventional Center of Valvular Heart Disease, Beijing Anzhen Hospital, Capital Medical University, No. 2 Anzhen Road, Chaoyang District, Beijing 100029, China

**Keywords:** Transcatheter aortic valve replacement, Membranous interventricular septum aneurysm, Case report

## Abstract

**Background:**

Membranous interventricular septum aneurysm (MISA) is a rare abnormality occurring in 0.3% of patients with congenital heart disease, which thereby increases anatomical complexity.

**Case summary:**

Transcatheter aortic valve replacement (TAVR) procedure was planned for a 71-year-old female patient from East Asia with a type 1 bicuspid aortic valve diagnosed with severe aortic stenosis by transthoracic echocardiography (TTE). Pre-procedural multidetector computed tomography (MDCT) clearly revealed an extremely horizontal aorta and a MISA originating from the sub-annulus with the upper edge extending 7.2 mm above the annulus. A probable communicating flow between the left ventricle and the aorta was confirmed by reviewing the TTE images. Moreover, there was a calcified raphe between the left- and right-coronary cusps. A downsized balloon-expandable valve (a 23 mm Sapien 3 valve with an additional 2 mL dilation) was therefore chosen and deployed with a 100/0 aortic/ventricular ratio position. The TTE post-implantation indicated a trace perivalvular leakage. The cardiac MDCT performed post-procedure, at the 6-month, and 12-month follow-ups demonstrated complete sealing and significant healing of the aneurysm.

**Discussion:**

Transcatheter aortic valve replacement utilizing a balloon-expandable valve was successfully performed for a case with membranous interventricular septum aneurysm extending above the annulus. Comprehensive imaging analysis before the procedure is crucial for TAVR with challenging anatomical conditions.

**Learning points**

- To describe the possibility of transcatheter aortic valve replacement (TAVR) in case with membranous interventricular septum aneurysm above annulus.
- To understand the anatomical characteristics of membranous interventricular septum aneurysm extending to the interleaflet triangle.
- To emphasize the significance of comprehensive imaging analysis including cardiac multidetector computed tomography and echocardiography in challenging TAVR cases.

## Introduction

Membranous interventricular septum aneurysm (MISA) is a rare condition, occurring in 0.3% of patients with congenital heart disease and in <0.1% of the general population. It is typically asymptomatic and can be categorized as true, false, or pseudo-aneurysm.^1^ Membranous interventricular septum aneurysm could be idiopathic or result from infection and trauma. While it is generally considered to have a benign clinical course, its standard treatment options remain inconclusive and not well established. In this case, we described a case with a MISA extending to the interleaflet triangle of the non-coronary cusp and right cusp above the annulus, which was extremely rare. Transcatheter aortic valve replacement using a balloon-expandable valve was successfully performed despite anatomical complexity derived from the MISA.

## Summary figure

**Figure:**
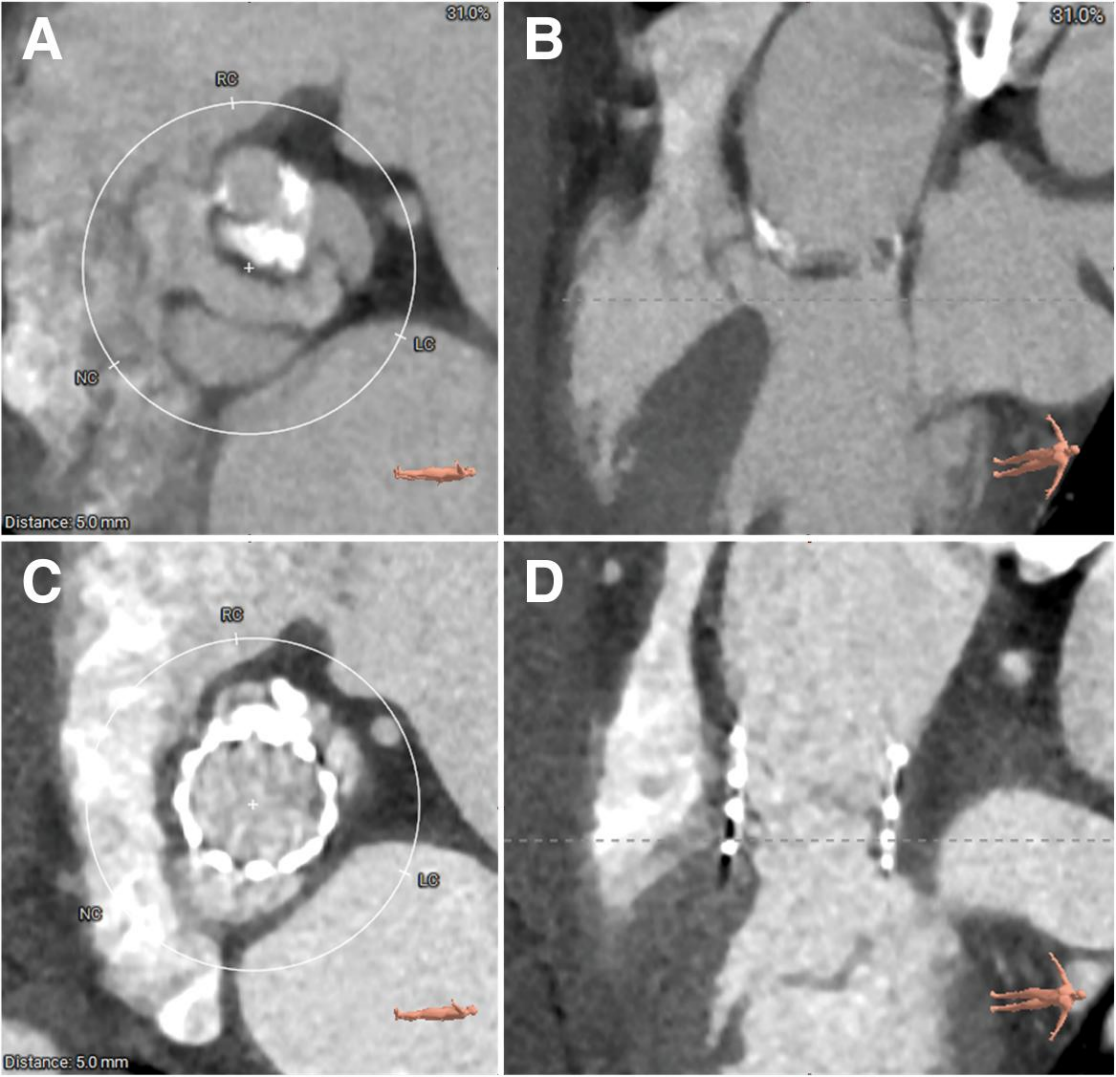


## Case presentation

A 71-year-old female patient presented with worsening exertional dyspnoea over the last 6 months. The past medical history of this patient included hypertension, type 2 diabetes, chronic obstructive pulmonary disease, and stress-induced gastritis. The physical examination revealed systolic ejection murmur (grade 4/6) and no abnormal lung sounds. Transthoracic echocardiography (TTE) revealed severe aortic stenosis (an aortic mean gradient of 70 mmHg, a peak velocity of 5.39 m/s), and left ventricular ejection fraction of 65%. Given a Society of Thoracic Surgeons (STS) score of 6.62%, the Heart Team deemed her intermediate risk for surgical aortic valve replacement and a transcatheter aortic valve replacement procedure was planned in consideration of patient preference, expected patient longevity, and valve durability.

**Figure 1:**
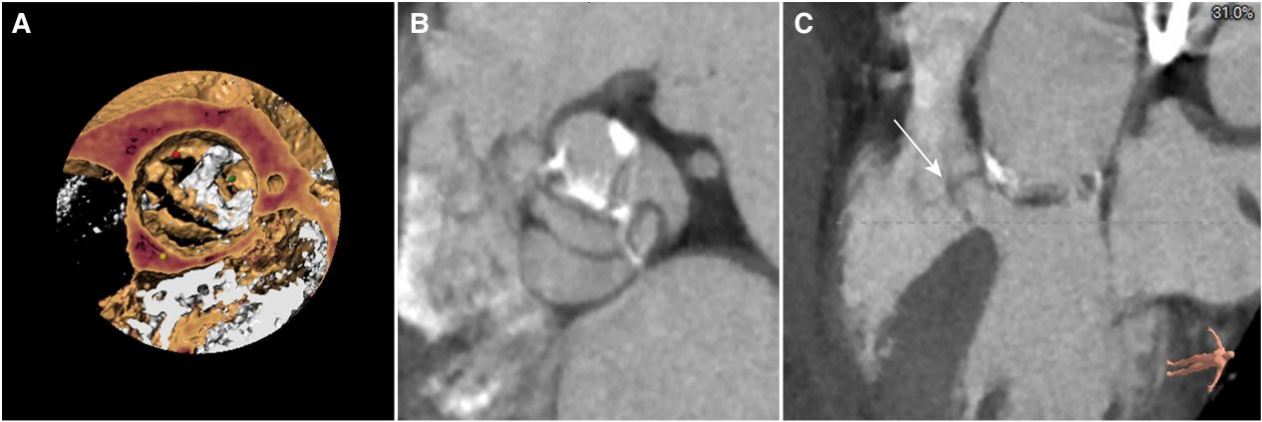
Multidetector computed tomography images of the aortic valve and septal aneurysm. (*A*) The type 1 bicuspid aortic valve in 3D view; (*B*) the type 1 bicuspid aortic valve with a septal aneurysm in the short view at a 7.2 mm height above the annulus; (*C*) the septal aneurysm originates from below the aortic annulus in the long view.

**Figure 2:**
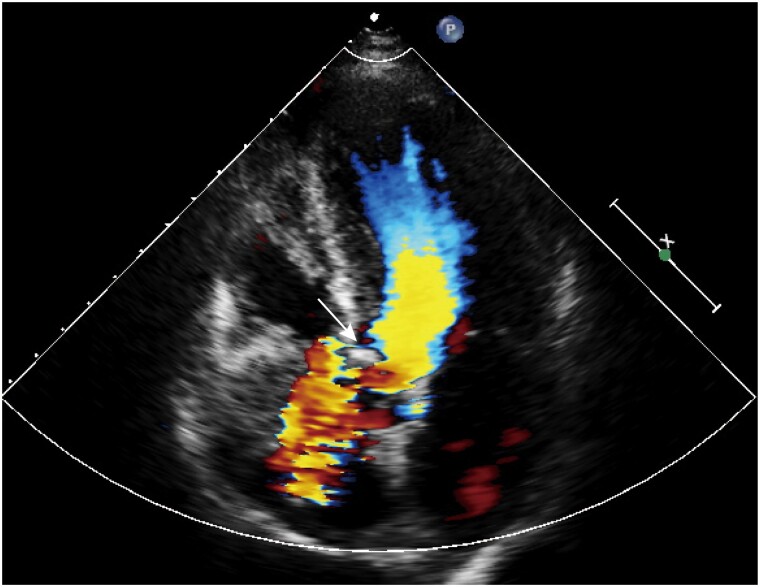
A Doppler flow imaging in the apical five-chamber view of the transthoracic echocardiography. Transthoracic echocardiographic image with colour Doppler flow showing the probable shunt (arrow) between left ventricular and aorta.

**Figure 3:**
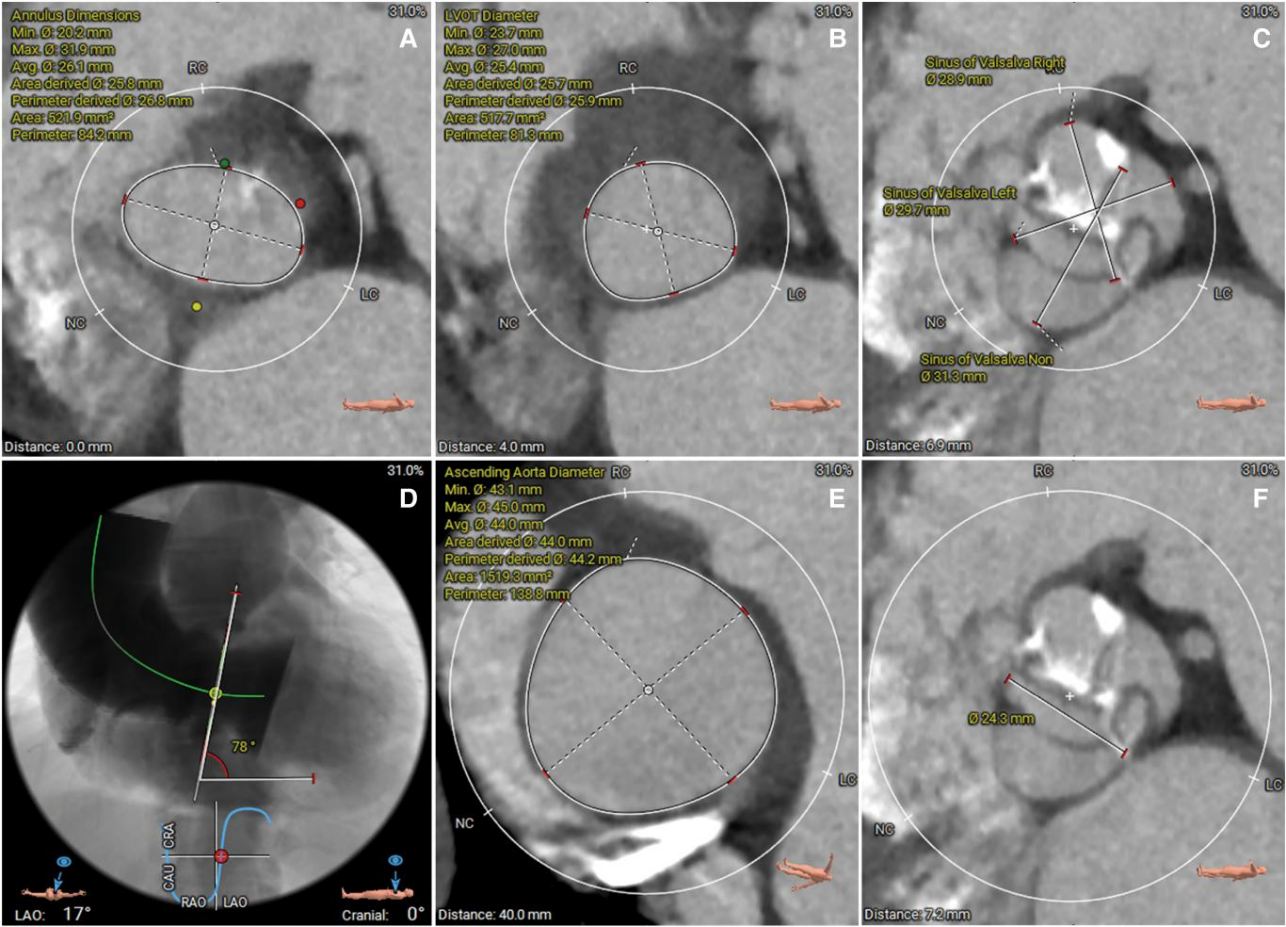
Basic assessment of the aortic valve. (*A*) Annulus measurement showing a perimeter derived diameter of 26.8 mm; (*B*) left ventricular outflow tract measurement; (*C*) sinus of Valsalva measurement; (*D*) significantly dilated ascending aorta with a maximum diameter of 45 mm; (*E*) horizontal aorta with a 78° aortic angle; (*F*) intercommissural distance measurement.

Pre-procedural multidetector computed tomography (MDCT) revealed a type 1 bicuspid aortic valve and a horizontal aorta with a 78° aortic angle. The Agatston calcium score was 1304, and there was a calcified raphe between left and right leaflets, oriented perpendicularly to the aortic wall, which elevated the risk of annular rupture. The CT clearly depicted a MISA originating from sub-annulus (*Figure 1*). The upper edge of the MISA extended 7.2 mm above the annulus involving the interleaflet triangle, which was out of the ordinary. A probable shunt between the left ventricle and the aorta across the aortic valve was observed when reviewing the TTE images (*Figure 2*). Basic measurements for TAVR are presented in *Figure 3*. Supra-annulus multiplane measurements were performed to enhance the understanding of the anatomy of this bicuspid aortic complex (see Supplementary material, *Figure 1*).

**Figure 4:**
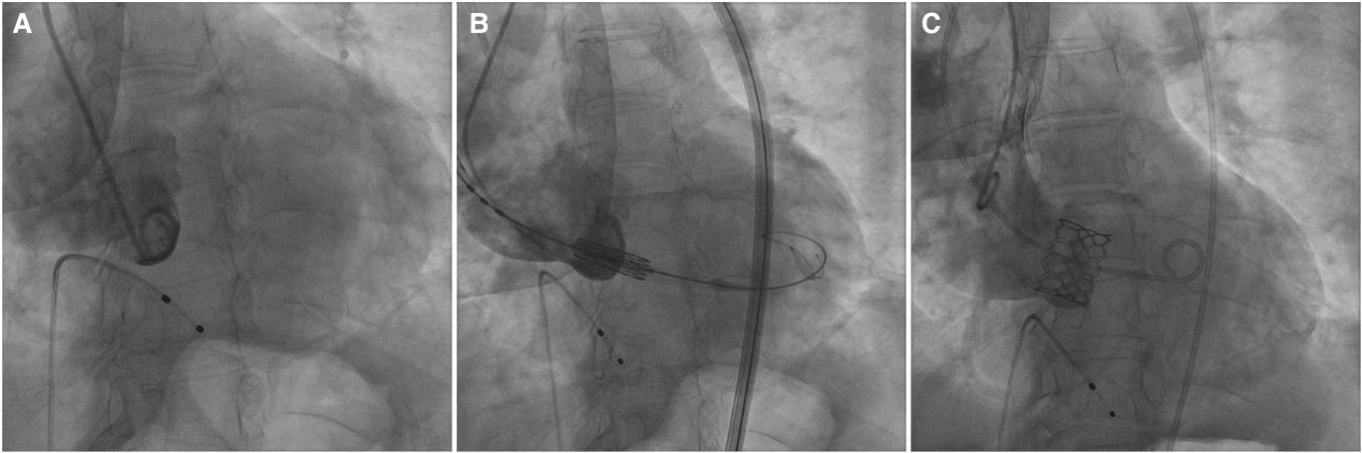
Implantation process of the 23 mm Sapien 3. (*A*) Aortic angiography before implantation in the coplanar view; (*B*) Sapien 3 positioning with the middle marker on the balloon 1–2 mm above the annulus, and the radiolucent line below the annulus; (*C*) final angiography after implantation, achieving the planned 100/0 ratio of valve stent in the aorta/left ventricular outflow tract.

Transcatheter aortic valve replacement with downsized strategy using a 23 mm Sapien 3 Valve with Commander Delivery System (Edwards Lifesciences, Irvine, CA, USA) was arranged, considering that the height of the upper edge of the MISA was higher than the 6.6 mm outer skirt sealing but lower than the 9.3 mm inner sealing height of the prosthetic valve. Nominal inflation volume plus an additional 2 mL in the balloon dilation was used to minimize perivalvular leakage (PVL). After meticulous positioning, the desired positioning was achieved with the bottom of the middle marker 1–2 mm above the annulus. Sapien 3 valve was deployed successfully under rapid pacing. Final angiography demonstrated an almost 100/0 ratio of valve stent in the aorta/left ventricular outflow tract (LVOT), with both coronaries unaffected (*Figure 4*). The TTE post-implantation showed mild paravalvular leak, with a peak velocity of 2.01 m/s and mean gradient of 3 mmHg. The patient was discharged on the third post-operative day.

**Figure 5:**
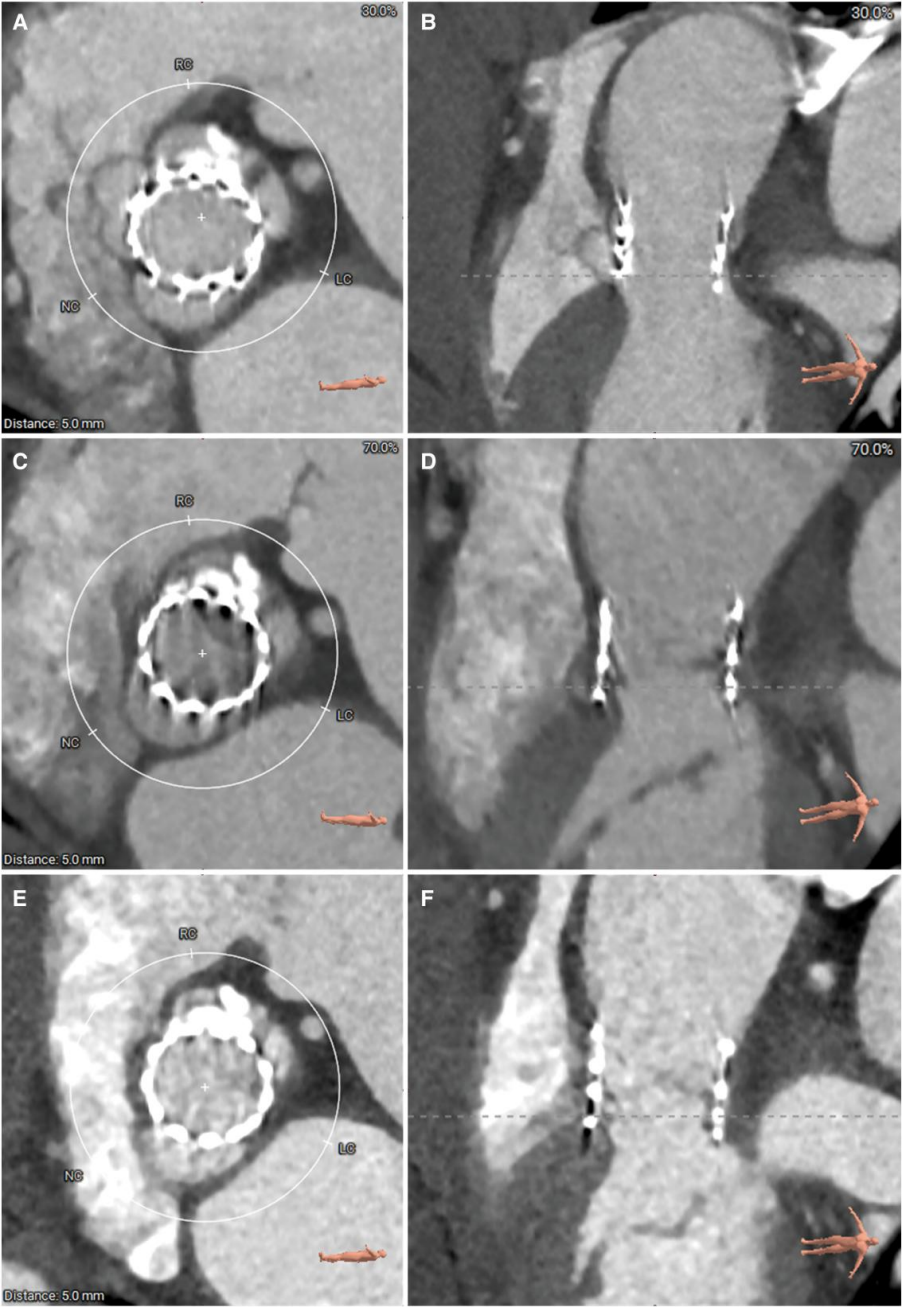
Cardiac computed tomography images of post-procedure, 6-month, and 12-month follow-ups. (*A* and *B*) Computed tomographic images of the interventricular membranous septum aneurysm and in the short view and in the long view post-procedure; (*C* and *D*) computed tomographic images at 6 months; (*E*) and (*F*) computed tomographic images at 12 months.

The patient reported significant symptomatic relief with no dyspnoea at 30-day follow-up and no systolic ejection murmur on physical examination. Transthoracic echocardiography conducted at 6-month and 12-month follow-ups revealed normal cardiac function and good function of the new bioprosthetic valve, with no evidence of any shunt between the left ventricle and the aorta. The cardiac MDCT was also performed at the same intervals after procedure for follow-ups, which indicated a substantial reduction in aneurysm size at 6 months, with further shrinkage and a near-complete resolution observed at 12 months (*Figure 5*). Additionally, the Kansas City Cardiomyopathy Questionnaire score improved markedly from 51.3 pre-procedure to 83.3 at the 12-month follow-up. The 6 min walking test also indicated a significant improvement in functional capacity, with the distance increasing from 180 m prior to the procedure to 320 m at the 12-month follow-up.

## Discussion

Based on current guidelines in which either SAVR or transfemoral TAVR is considered appropriate and a bioprosthesis is preferred over a mechanical valve among patients aged >65 years with an intermediate risk for surgery,^2^ a TAVR procedure was planned for this 71-year-old female patient after the discussion with the Heart Team and patient. The calcified raphe between the left and right leaflets in our case significantly increased the risk of aortic rupture when using balloon-expandable valve during TAVR procedure.^3^ However, the extremely horizontal aorta might hinder the ideal positioning and increase dissection risk, making the use of a self-expanding valve even riskier.^4^ While the severity of calcification is an established risk factor for annular rupture, the anatomical location and morphological characteristics of the calcification are also pivotal in assessing rupture risk. In our case, the calcification, though not severe, was situated in the raphe between the right and left coronary cusps and oriented perpendicularly to the aortic wall, exhibiting a sharp configuration that could potentially perforate the annulus during balloon dilation. Deploying a 26 mm Sapien 3 Valve inflated with standard volume for TAVR could significantly increase the risk of aortic rupture, making a downsized approach more prudent. Furthermore, when confronted with the choice between under-expanding a 26 mm Sapien 3 Valve and over-expanding a 23 mm Sapien 3 Valve, we preferred the latter, utilizing nominal inflation volume with an additional 2 mL, in order to prevent improper expansion and to enhance durability of the valve prosthesis. A 23 mm balloon-expandable valve with an excessive expansion was therefore chosen for this patient based on the risk balance between the rupture and PVL. Comprehensive imaging analysis, utilizing MDCT and echocardiography, plays a crucial role in decision-making and device selection in this case, helping to avoid complications and optimize procedural outcomes.

The MISA is usually regarded as a clinical course of no particular significance and is always located below the aortic valve. Membranous interventricular septum aneurysm with upper edge exceeding above the annulus rather than a subvalvular aneurysm, as we described in our case, is considerably uncommon.^5^ The TTE images indicated a probable aorta-to-ventricle shunt across aortic valve. CT could provide more conclusive and accurate information about the size and location of the aneurysm, as well as delineate the relationship between the membranous septum aneurysm and the aortic valve. In our case, the extension of the MISA above the annulus to the interleaflet triangle increased the risk of residual aortic regurgitation if the aneurysm was not entirely sealed and the leaflets were pulled apart by the device.

In consideration of the aortic insufficiency attributed to the continuation of the MISA into the aortic cusps, we tried to achieve adequate aneurysm sealing when selecting and deploying the prosthetic valve. We therefore used a device with skirt and performing a higher implantation. In regular TAVR cases, the ideal position would be conventional 80/20 ratio of valve frame in the aorta/LVOT or 90/10 to reduce conduction abnormalities and permanent pacemaker requirement.^6^ In our case, the membranous aneurysm made it difficult to seal at the annulus plane due to upper edge of the MISA neck extending 7.2 mm above the annulus. A strategy of 100/0 aortic/ventricular ratio position was chosen to ensure sufficient inner skirt sealing at the interventricular membranous septal aneurysm. The final angiography and CT post-procedure revealed that the bottom of the Sapien 3 valve was equal to base of the non-coronary cusp and 3–4 mm lower than the base of the left- or right-coronary cusp.

The CT post-procedure confirmed that the MISA was sealed by the prosthetic valve. The sealing capacity was also confirmed by the fact that the aneurysm significantly shrank after procedure. A previous report about a challenging case of pure aortic regurgitation also demonstrated complete healing of the MISA that originally extended into the aortic annulus at 2 years after TAVR procedure using a self-expanding valve.^7^

In conclusion, our case demonstrated the feasibility of TAVR procedure in patients with MISA communicating with aortic root the risk of valve mispositioning. Transcatheter aortic valve replacement could be considered as an alternative for high- and intermediate-risk patients in spite of complex heart structure. Comprehensive imaging analysis including cardiac MDCT and echocardiography is of supreme importance for procedure planning and valve selection in TAVR cases with challenging anatomies.

## Supplementary Material

ytae523_Supplementary_Data

## Data Availability

The data underlying this case report will be shared on reasonable request to the corresponding author.

## Lead author biography

Professor Song Guangyuan, the Director of the Heart Valve Intervention Center of Beijing Anzhen Hospital, Capital Medical University. In 2015, as the first professional in China to complete post-doctoral training in interventional diagnosis and treatment of valvular heart disease in the USA, he is an expert at the forefront of interventional treatment of structural heart disease and complex coronary artery disease. He has led or participated in over 20 clinical research projects related to interventional treatment of structural heart disease and complex coronary artery disease.

## Notes

**Consent:** The authors confirm that written consent for submission and publication of this case report including images and associated text has been obtained from the patient in line with COPE guidance.

**Funding:** None declared.
