# Supplementary material for: Transcatheter aortic valve replacement in a bicuspid aortic valve with membranous interventricular septum aneurysm communicating with aortic root: a case report
Source: Eur Heart J Case Rep. 2024 Oct 4;8(10):ytae523. doi: 10.1093/ehjcr/ytae523 (PMC11465161; doi:10.1093/ehjcr/ytae523)
Supplement: ytae523_Supplementary_Data [file ytae523_supplementary_data.docx]

**Figure 1** Multiplane measurements for bicuspid aortic valve.


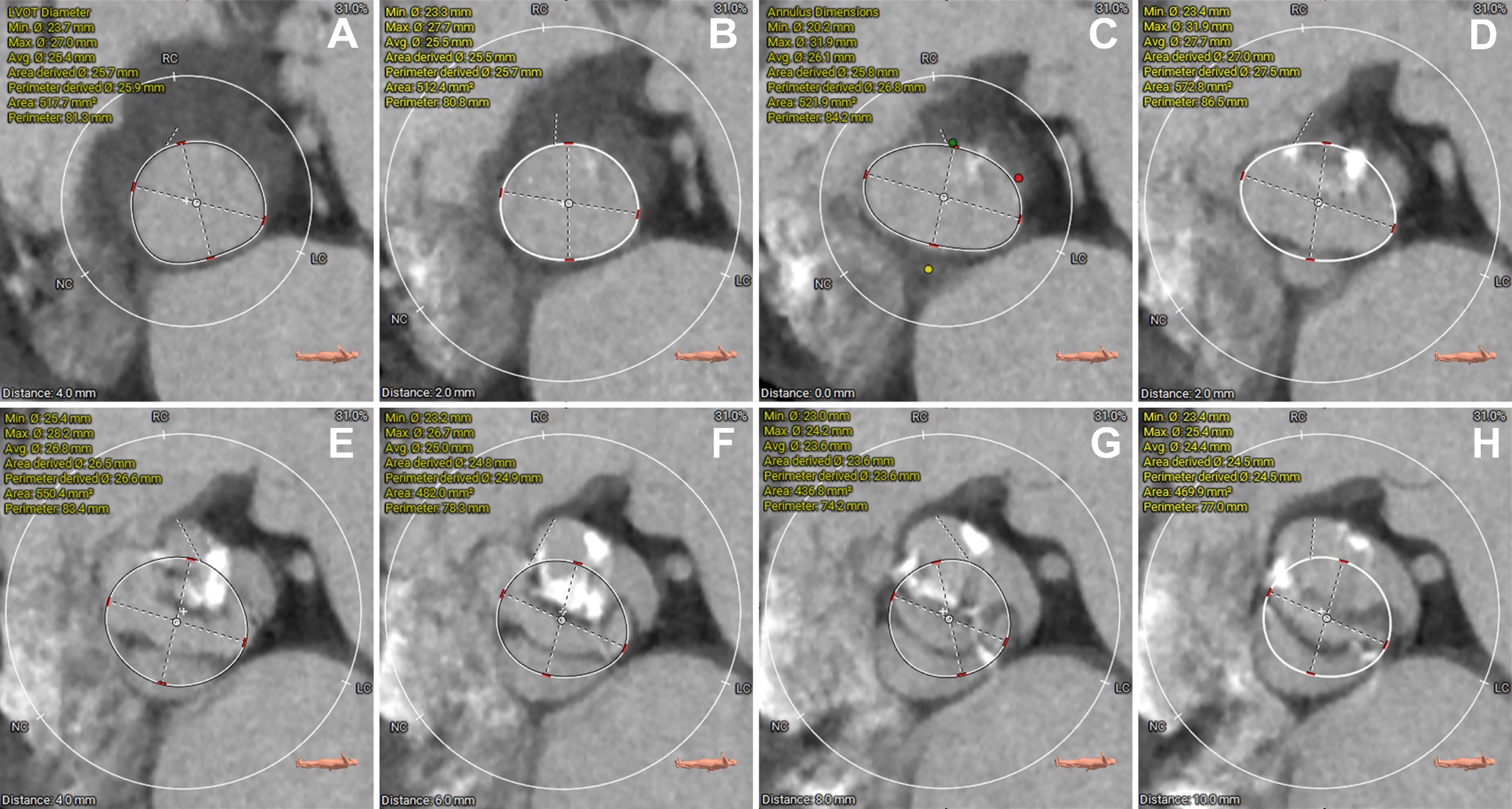


Multiplane measurements from left ventricular outflow tract to 10mm above the annulus at a 2 mm interval.
